# Immunosenescence and Inflamm-Aging: Clinical Interventions and the Potential for Reversal of Aging

**DOI:** 10.7759/cureus.53297

**Published:** 2024-01-31

**Authors:** Samayak J Kumar, Samarth Shukla, Sunil Kumar, Preeti Mishra

**Affiliations:** 1 Pathology, Jawaharlal Nehru Medical College, Datta Meghe Institute of Higher Education and Research, Wardha, IND; 2 Medicine, Jawaharlal Nehru Medical College, Datta Meghe Institute of Higher Education and Research, Wardha, IND

**Keywords:** pathogenesis, intervention, aging, immunosenescence, inflamm-aging

## Abstract

Inflammation is often associated with the impairment of the ability to sustain the consequences of the physical, chemical, nutritional, and antigenic triggers of inflammation. The process of immunosenescence may only partially be explained by the senescence of cells, tissues, or the organism, and, hence, the hallmarks of immunosenescence may be markedly and differentially affected by the history of an individual's pathogenic encounter. Inflammation is a key component of immunosenescence, which itself is a direct consequence of aging. This review article highlights the therapeutic interventions for slowing the processes of inflamm-aging and immunosenescence and the possible reversal of aging and includes domains of immunomodulatory interventions, vaccination strategies, nutritional interventions, stem cell therapies, personalized medicine, microbiome interventions, and the positive effects of physical activity and exercise.

## Introduction and background

Natural killer (NK) cells usually have preserved cytotoxic function, with an associated increase in cell count as a hallmark of aging. On the other hand, neutrophils may be associated with decreased efficacy in bacterial phagocytosis and oxidative burst. Aging is often associated with the impairment of coping mechanisms of body tissues to the damaging effects of physical, chemical, nutritional, and antigenic triggers of inflammation, a phenomenon called inflamm-aging. Prolonged low-grade and chronic inflammation may often be associated with tissue degeneration and impairment of normal tissue function.

The T- and B-cell responses tend to differ quantitatively as well as qualitatively along with the progression of age, which may have a direct consequence on the efficacy of the immune response and its decreased intensity against newly encountered antigens. In older individuals, this consequence of aging may manifest as a decreased ability to respond to novel antigens and even to vaccines, the consequence of which is an increased susceptibility of such individuals to infections. Individuals with a reversal of the CD4/CD8 T-cell ratio, a suppressed immunological response against mitogenic stimuli, and a severely reduced B-cell count were all associated with decreased life expectancy, all of which are essential parts of a greater spectrum of immunosenescence. The hallmark features of immunosenescence are (i) a decreased immunologic response against novel antigens; (ii) the accumulation of memory T-cells because of repeated infections; and (iii) the presence of a chronic low-intensity inflammation termed "inflamm-aging.” The process of immunosenescence may only partially be explained by the senescence of cells, tissues, or the organism, and, hence, the hallmarks of immunosenescence may be markedly and differentially affected by the history of an individual's pathogenic encounter [1].

With aging, the immune system fails to respond to chronic stimuli of antigen-presenting cells for a prolonged period, which may be followed by an upregulation of acute phase proteins and pro-inflammatory cytokines. A low-level inflammatory state is supported and maintained by the oxidative stress accompanied by aging, which itself acts as a catalyst for further oxidative stress [2]. The leading cause of inflammation is the activation of various signaling networks, including those regulated by the enhancer of transcription factor of activated B-cells (NF-κB), which may aid critically in inflammatory processes, especially when combined with stimuli such as the presence of mitochondrial DNA in circulation, obesity, senescent cells, gut microflora, and dietary triggers. The theory of oxi-inflamm-aging suggests the prolonged effects of oxidative stress upon immune cells, particularly on regulatory systems such as endocrine, neural, and immune, including the mutual interactions amongst them [3].

The true potential of the research on immunosenescence and inflammation hasn’t yet been tapped. Several aspects of inflammation, such as mechanisms, biomarkers, research models, and interventional technologies, have not yet been elucidated. Moreover, the study of inflammation requires an extensive field of research investigations due to its involvement in cells, organs, and even the body as a whole [4]. Targeted therapies for aged immune cells, like rejuvenating aged immune cells with the use of induced pluripotent stem cells (iPSCs) [5], are still being researched. The domain of regenerative medicine includes the use of cell and gene therapy and tissue engineering in the rejuvenation of thymic tissue and holds the potential to provide a different outlook on the reversal of aging.

Search methodologies

**Figure 1:**
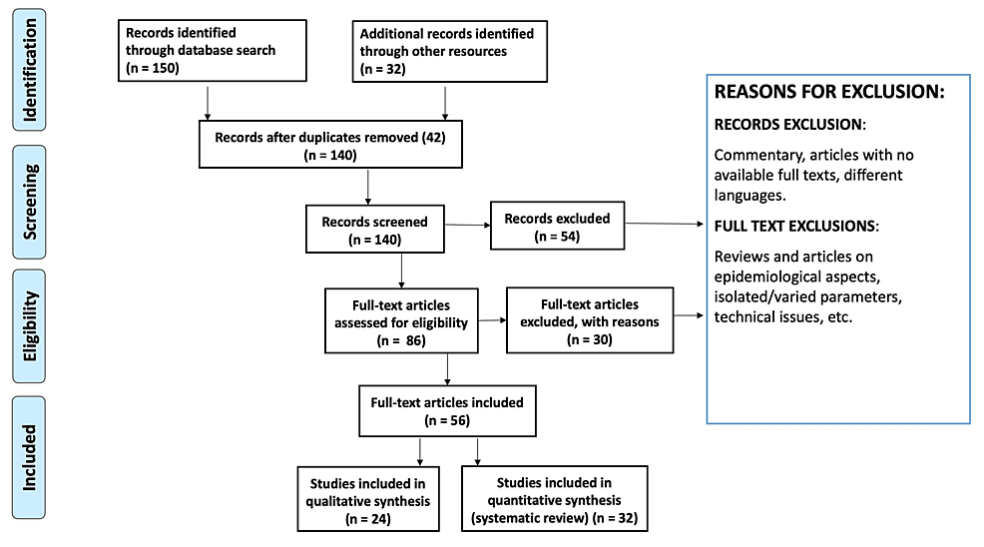
PRISMA chart of included studies PRISMA: Preferred Reporting Items for Systematic Reviews and Meta-Analyses

Extensive literature research was conducted using Medline, PubMed, Cochrane Library, and Google Scholar using keywords like immunosenescence, inflamm-aging, and age reversal. The research material was screened for relevance by title, while several articles were excluded from the research because they were either not complete, in a foreign language, or duplicates. Articles that showcased the factors that could potentiate the reversal of immunosenescence and inflamm-aging were included in the scope of the article. Firstly, the identified studies were screened by the title and abstract of the article. A full-text examination was followed up to further screen the articles. The researchers came to a decision in case of any conflict by exploring the potential of any variables in the study choice. Figure 1 showcases the flow of literature search by the Preferred Reporting Items for Systematic Reviews and Meta-Analyses (PRISMA) method of literature search. Data like the name of the authors and the year of publication, demographics of the research population, definitions for immunosenescence and inflamm-aging, and the conclusion and key findings were collected for all research references.

## Review

Inflamm-aging is a key component of immunosenescence, which itself is a direct consequence of aging. The “remodeling” nature of the mechanism of inflamm-aging aids it in combating the adversities faced by an organism as a consequence of general aging. This mechanism of adaptive remodeling is in association with a series of primary and secondary factors, commonly known as immunobiography [6,7].

Pathogenesis of immunosenescence and inflamm-aging

**Figure 2:**
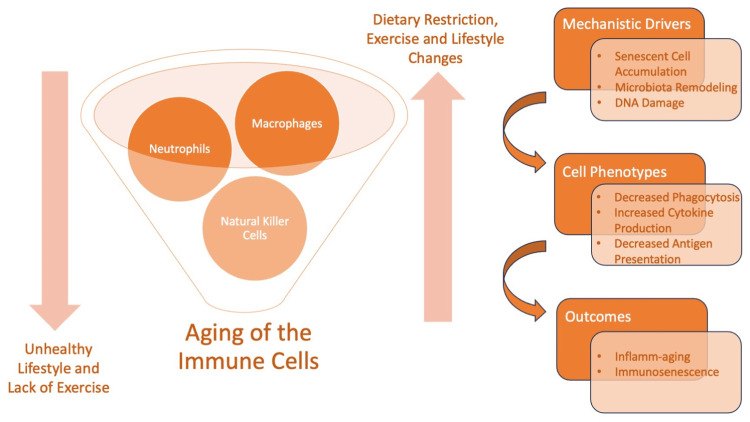
Factors responsible for aging of the immune system Image Credit: Author

Immunosenescence is a natural consequence of aging, which may be further accelerated by factors like cellular senescence and chronic inflammation [8]. Further contributing factors like pollution, radiation, and a lack of exercise can aggravate the effects of immunosenescence and inflamm-aging. The accumulation of dysfunctional macromolecules and host-derived cell debris of endogenous origin due to their enhanced production and/or impaired or inadequate elimination is a major cause of inflamm-aging and leads to chronic tissue damage [9]. Transcription factors, such as the MYC seen in the thymus of aged rats, are associated with immunosenescence and related to the differentiation and activation of T-cells [10]. A very common consequence of chronic antigen load is that it eventually leads to the pooling of immunological space by an excess of late-differentiated T-lymphocytes [11]. Studies on bone marrow cells carried out by Pangrazzi et al. [12] indicated that the molecular expression of substances required for the continual maintenance of the memory T-cells and plasma cells in the bone marrow is altered with the process of aging. Figure 2 highlights the possible factors responsible for the aging of the immune cells.

DNA Changes in Immunosenescence

All biological processes are regulated by epigenetic mechanisms responsible for controlling transcription and translation reactions. Epigenetic mechanisms controlling gene transcription and translation are responsible for the regulation of all biological life processes. Dysregulation of these mechanisms, such as histone modifications and non-coding RNAs, is usually identified and is considered a major hallmark of aging, along with an associated increased risk of tumors and autoimmune disorders in such individuals [13]. Aging has been shown to greatly impact the transcriptional landscape of cells, mostly neutrophils and dendritic cells [14]. Dysfunction of neutrophils and macrophages as a consequence of aging most contributes to overall immune dysfunction with marked changes in chemotaxis, phagocytosis, microbicidal action of leucocytes, and intracellular granule secretion [15,16]. Animal studies by Benayoun et al., including those conducted on the primary bone marrow of mice, showed transcriptional changes in neutrophils vastly influenced by organismal aging [17].

Therapeutic interventions

Immunomodulatory Interventions

A major factor contributing to the imbalance of immune cell populations, especially for T-cells, is thymic involution, which occurs gradually through early childhood and puberty. Thymic tissue can be made to fall into two major classes: the epithelial tissue and the perivascular space of non-epithelial origin, without the presence of thymopoiesis [18]. Thymic atrophy leads to a fall in the naïve T-cell population, an upregulation of late-differentiated memory T-cells, and the inability of these cells to translocate to the peripheral tissues as a result of epithelial tissue collapse and the simultaneous increase of perivascular space that gradually occupies the entirety of the elderly thymus [19]. Recent research, such as that conducted by Sauce et al. [20], concluded the prevalence of premature immunosenescence in young adults who underwent thymectomy very early on due to the presence of congenital heart diseases. These findings were comparable to those of elderly individuals with altered T-cell profiles, which provides relevant evidence of the relationship between thymic involution and immune aging. Research into thymic regeneration by stem cell therapies and administration of growth hormone and its role in the reversal of immunosenescence is still in the early stages but may be a boon for future studies.

Senolytic drugs are being used as pharmacological strategies to improve immune responses in young geriatric age groups. These drugs induce apoptosis in damaged senescent cells selectively while sparing healthy cells, and they have the potential for the treatment of age-related chronic conditions. Among them are dasatinib, a tyrosine kinase inhibitor, and quercetin, a flavonoid drug, which can be used for the targeted elimination of senescent adipocyte progenitors and endothelial cells, respectively [21,22]. Low-dose administration of cyclophosphamide in a study by Scurr et al. [23] provided evidence of depletion of regulatory T-cells that were used against colorectal cancer. These interventions can similarly be replicated for enhanced immune responses in the elderly.

Vaccination Strategies

Older adults (>65 years) represent the most vulnerable group, which sorely lacks appropriate vaccination strategies like adjuvant vaccines capable of eliciting immune responses. Lack of proper vaccination, paired with the onset of immunosenescence and inflamm-aging puts them at a much higher risk of developing infections due to their inability to develop appropriate immune responses against the presenting antigens. Improvement of immunity in such a population requires approaches like enhancing the antigenicity of the vaccine by increasing the antigen dose or by the use of adjuvants combined in the vaccine formulations [24]. The benefits of the use of senolytic and other immunomodulatory drugs have been highlighted in several recent studies for improved vaccine responses [25]. The use of modified adjuvants in the efficacious combat of immunosenescence to produce an enhanced response is required to counter the deficiencies of immunity. Combating immunosenescence effectively requires the modification of adjuvants to stimulate robust responses, which may aid in overcoming the deficiencies of immunogenicity. Studies of novel adjuvants mostly focus on the stimulation of higher antibody responses coupled with a greater T-cell activation rate and their impacts on diseases in older adults.

The most prominent examples among these include studies of influenza vaccination and increasing its antigenicity in the elderly [26,27]. One way of accomplishing this could be by using emulsion-based adjuvants, which were shown to elicit a higher antibody response in the elderly population and were also found to be cross-reactive to heterologous viral strains [28]. Another way of overcoming diminished responses could be by increasing the amount of haemagglutinin administered per dose [29]. Similar studies of the implementation of adjuvants for vaccines like herpes zoster, pneumococcal tetanus, and diphtheria vaccines were conducted and found to be effective in elderly symptomatic patients [30].

Nutritional Interventions

Diet can dynamically influence the maturation and effector responses of the immune system. A healthy nutritional exposure in the early stages of life is key to the development of immunocompetence against pathogens and disorders with progressing age [31]. Phytochemicals that are food-based are, in fact, great sources of immunomodulators, including immunosuppressants and stimulants. Garrigue et al. conducted a study on Sprague-Dawley rats and observed that the long-term consumption of resveratrol, a plant-based biochemical, enhanced neurocognitive performance by suppressing inflammatory pathways [32]. Polyunsaturated fatty acids (PUFAs), particularly n-3 PUFAs like eicosapentaenoic acid and docosahexaenoic acid, play an essential component of a balanced diet and are associated with numerous positive health effects, including protection against chronic modifiable diseases like cardiovascular disease, chronic inflammatory disease, diabetes, and age-related cognitive dysfunction [33,34]. Some studies have proven lengthening of the telomeres of leucocytes is associated with the consumption of n-3 PUFAs in elderly individuals, leading to an attenuation of multiple markers of immunosenescence [35]. Even in middle-aged adults, PUFAs lead to a decrease in pro-inflammatory markers like cytokines, which ultimately reduces systemic inflamm-aging [36].

Stem Cell Therapies

Bone marrow stem cells, which give rise to all the immune cells, undergo a gradual decline in number associated with a generalized decline of the total bone marrow hematopoietic tissue with progressing age [25]. These aging-associated alterations include self-renewal and myelopoiesis-favored differentiation [37]. An increased risk of leukemogenesis is often associated with aging and inflammatory processes; hence, the elimination of unwanted factors of inflamm-aging may help preserve stem cells as well as immune functions. This measure may decrease the prevalence of malignant clones and prevent a functional decline of hematopoiesis; however, inflamm-aging processes at these molecular, cellular, and tissue levels may require further investigation [38]. There has been a lack of studies into the potential rejuvenation of aged stem cells in bone marrow and thymic tissue. Such stem cell therapies, including hematopoietic stem cell (HSC) transplantation in animal models, have found little success [39].

Microbiome Interventions

The impact and relevance of the composition of the commensal microflora of the gut in immune homeostasis are markedly prominent [33]. The dysbiotic gut microbiota is a key component of various inflammatory and immunological disorders, like immunosenescence [40]. Several studies have concluded the efficacy of probiotics in modulating aspects of immunosenescence. A recent study by Balcells et al. [41] reported the findings of improved functional and cellular markers of immunosenescence and the restoration of age-related degeneration of the thymic medulla. Reported anti-immunosenescence effects of probiotics through randomized controlled studies on humans also played a crucial role in their understanding. One such study by Castro-Herrera et al. [42] observed an enhanced response to seasonal influenza vaccination in elderly subjects after a 12-month-long therapy with a combination of various probiotics such as Lacticaseibacillus and Bifidobacterium. Some species of Lactobacilli and prebiotic corn fiber given to healthy elderly individuals also resulted in a markedly increased NK cell response while suppressing systemic inflamm-aging [43].

Interventions based on individual biomarker profiles to address specific immune deficiencies and inflammation markers have proven to be effective in limiting or even reversing the effects of immunosenescence. Increased plasma levels of interleukin-6, interleukin-1, and TNF-α associated with aging may be influenced by an underlying overactivation of the immune system as a consequence of a chronic sub-inflammatory condition. These can be considered biomarker-guided treatment optimization strategies [44,45].

Exercise and Physical Activity

Several studies have indicated the role of physical exercise in the slowing of immunosenescence and reversing associated molecular alterations. Acute exercise like a single session of treadmill running was found to upregulate telomerase activity and longer telomere length, which are essential for healthy cell division [46]. Increased morbidity and mortality from diseases may be associated with sedentary behavior accumulated over weeks, months, or even years due to several immunological and inflammatory alterations [47]. Acute and frequent bouts of moderate- and high-intensity workouts are shown to alter and enhance several aspects of immune function [48,49].

Yoga and yogic asanas, including standing (tadasana and urdhva hastasana), sitting (paschimottanasana), and controlled breathing exercises like pranayama, have been shown to improve blood supply and strengthen muscles. Yogic interventions such as these have been proven to be effective in the control of diabetes and hypertension, which can further have a protective role against chronic conditions like chronic kidney disease (CKD). Further, studies have shown improved antioxidant activity and decreased oxidative stress in individuals practicing yogic exercises in patients with CKD and hypertension [50,51].

Mind-Body and Stress Reduction Interventions

Psychological stress may induce inflammatory activity, which has deleterious effects on the body, especially in the early stages of development. A correlation can be drawn between immune system activation and psychological processes; the study of the same being done under psychoneuroimmunology can help in the better understanding of the effects of psychosocial stress factors on aging. The release of proinflammatory cytokines as a result of immune activation may induce changes in mood and behavior. Conversely, mental disorders like depression and post-traumatic stress disorder have been shown to alter immune function. Models of acute stress, including studies such as those conducted by Boyle et al. [52], have been correlated with effective activation of the innate immune system, while studies linked with the study of chronic stress in individuals have been used to better understand the neuroimmune pathways relevant to immunosenescence and aging mechanisms. Important study fields also include environmental stress factors that are directly or indirectly correlated with poor health.

Interventions focusing on improvements in quality of life and well-being have led to a reduction in psychological stress levels and overall positive health effects. Mindfulness interventions demonstrated by studies such as those conducted by Bower et al. [53] have been shown to modulate gene expression linked to a decrease in inflammatory activity and, hence, provide proof that counteracting negative states like loneliness may be an important intervention pathway against inflammation. Pharmacological interventions targeting inflammation and stress reduction have only found limited success but continue to pose immense scope in the field. Anti-inflammatory agents have been shown to have positive effects on depression. Integration of immune system-enhancing therapies with mind-body and behavioral interventions can be targeted to slow aging processes and counteract immunosenescence and inflammatory processes.

Ayurvedic Interventions

Ayurvedic interventions embrace the inevitability of the aging process and harmonize with nature. Healthy aging, according to Ayurveda, requires the induction of healthy lifestyle practices while encouraging healthy mind and body transformation. Large-scale controlled trials on such interventions have been limited due to the lack of safety and efficacy of trials, but research into this domain possesses potential. The concept of “healthy conscious eating” or “Ahara Vihara” institutes the importance of a healthy and timely diet for a disease-free lifespan [54]. Additionally, Ayurveda also emphasizes sleep and healthy routine practices due to genetic changes associated with the biological clock and circadian rhythm and their role in inflammatory processes. Panchakarma therapies involving the use of herbal medications, a vegetarian diet, and meditation yielded significant alterations in total levels of glycerophospholipids and sphingolipids, along with a boost in the host immune responses [55]. Regular oil massage, or "Abhyanga,” with warm oil boosted with specific herbs has been shown to delay age-related and pathological changes in an individual, especially changes involving the central nervous system due to an increase in cerebral blood flow and an increase in circulating lymphocytes [56]. Table 1 summarizes the studies by various authors on the pathogenesis and therapeutic interventions of the aging of the immune system.

**Table 1:** Study conclusions by various authors pertaining to the pathogenesis and clinical interventions of immunosenescence and inflamm-aging iPSCs: induced pluripotent stem cells

S. no.	Author and year	Conclusion of the study
1.	Aiello et al., 2019 [1]	Inflamm-aging paired with an age-associated increase in memory T-cells and a simultaneous decrease in new leucocytes in the peripheral blood are hallmarks of immunosenescence, which may be modulated by dietary and immunomodulatory interventions.
2.	Martínez de Toda et al., 2021 [2]	The rate of aging and the lifespan of an individual can be modulated by manipulating the immune cell function, which can furthermore be achieved by affordable lifestyle strategies and nutritional intervention.
3.	De la Fuente et al., 2009 [3]	The basis of age-related impairment of the immune system is oxidative and inflammatory stress conditions accompanied by the activation of NF-κB in these cells.
4.	Xia et al., 2016 [4]	Diseases like atherosclerosis, diabetes, and cardiac disorders are evidently associated with the processes of immunosenescence and inflamm-aging, which tend to occur together and complement each other.
5.	Lapasset et al., 2011 [5]	The iPSCs may have applications in tissue regeneration therapies acting as a form of regenerative medicine for aged individuals by the reversal of telomere shortening, oxidative stress, and gene expression profiles.
6.	Franceschi et al., 2017 [6]	The study of immunobiography could help in the understanding of the effects of immunosenescence on immune responses against vaccines and pathogens.
7.	Franceschi et al., 2018 [7]	Biomarkers like DNA methylation and genomics could help in accessing and reversing the accelerated aging observed as a manifestation of inflamm-aging.
8.	Accardi et al., 2018 [8]	The connection between immune-inflammatory responses and nutrient-sensing pathways has been proven to be associated with longevity and delay of age-related diseases.
9.	Feldman et al., 2016 [9]	The release of DAMPs as a consequence of oxidative stress leads to the initiation of inflammatory processes that accelerate aging processes.
10.	Sidler et al., 2013 [10]	A variation in the number of affected genes was noticed in the mRNA transcript profile of splenic and thymic tissue of rats with varied ages and tissues.
11.	Lu et al., 2022 [11]	Innate immune cells are prone to age-related dysfunction in many of their key phenotypes, which is a hallmark of Immunosenescence, which may take place due to remodeling of their transcription programs.
12.	Pangrazzi et al., 2017 [12]	The implications of inflammation and oxidative stress in inflamm-aging make antioxidants play a key in the improvement of immunological memory thereby counteracting immunosenescence.
13.	Liu et al., 2023 [13]	An increased proliferative capacity and tumorigenic potential may be achieved for cells re-entering the cell cycle by the reversal of the senescent cellular state.
14.	Lai et al., 2019 [14]	Understanding the aging transcriptome is crucial in the identification of regulatory targets which could be useful in the slowing down or reversal of aging and extension of a healthy lifespan.
15.	Wenisch et al., 2000 [15]	A reduction in the bactericidal action, phagocytosis, and chemotaxis of neutrophils was observed in close association with senescence.
16.	Tseng et al., 2012 [16]	The impaired ability of an elderly human host in the successful clearance of invading pathogens may contribute to the development of invasive methicillin-resistant S. aureus infection. This can be used to demonstrate the relatively easy dissemination of pathogens in elderly subjects.
17.	Benayoun et al., 2019 [17]	Analysis of specific epigenomic and transcriptomic landscapes can be performed to predict transcriptional changes during aging, providing necessary insight into the mechanism of immunosenescence.
18.	Lu et al., 2021 [18]	A study of neutrophils and the identification of their characteristics across varied ages and sexes in mouse can be done for the targeted improvement of immune responses.
19.	Palmer, 2013 [19]	Thymic involution is amongst the most characteristic features of immune aging and may be heavily influenced by extrinsic and intrinsic factors like infections, pregnancy, and early life events.
20.	Sauce et al., 2009 [20]	Thymectomy of individuals in their early childhood has been demonstrated to develop decreased absolute CD4+ and CD8+ counts with the progression of age, aiding to increased risks of infections and increased immune cell death.
21.	Crooke et al., 2019 [21]	Senile changes in a single cell subset can lead to the compromise of the entire immune system due to its intricacy, hence the severity of reduced response of vaccines in the elderly which must be compensated for.
22.	Zhu et al., 2015 [22]	Targeting pro-senescence and anti-apoptotic factors with senolytic drugs leads to the selective killing of senescent cells. Periodic administration of these drugs has been shown to enhance lifespan and reduce frailty.
23.	Scurr et al., 2017 [23]	A significant antitumor activity was observed in the case of decreased levels of regulatory T-cells in metastatic colorectal cancer, which was achievable by the administration of cyclophosphamide, hence providing scope for future anti-cancer medications.
24.	Weinberger, 2018 [24]	Different adjuvants and relevant antigenic stimuli were tested to study the varied immunological signatures so obtained, which can further be accessed to develop novel vaccines for the elderly.
25.	Kirkland et al., 2017 [25]	Targeting cellular senescence via therapies like senolytic drugs may be key in the reversal of fundamental aging mechanisms like senescence in mammalian species.
26.	Franco et al., 2013 [26]	Genetic variation associated with antigen processing and membrane trafficking plays an important role in vaccination response.
27.	Tan et al., 2014 [27]	Demonstration of the gene set enrichment method and its use in recording subtle transcription changes as an approach to a better understanding of the immune response.
28.	Del Giudice et al., 2006 [28]	The use of adjuvants with classical influenza vaccination produced antibody responses in >98% of individuals against the usual 76-80% responses in vaccines without adjuvants.
29.	DiazGranados et al., 2014 [29]	A higher dose of an influenza vaccine produced clinically greater antibody responses in 65-year-old affected individuals against influenza compared to its standard-dose counterpart.
30.	Pereira et al., 2020 [30]	As a counter to inflamm-aging, which has been shown to inhibit adequate immune responses against vaccines in the elderly, alternative approaches like increasing the vaccine dose, use of adjuvants, or alternative routes of administration can be beneficial.
31.	Tourkochristou et al., 2021 [31]	Several nutritional factors and dietary interventions have proven to affect molecular pathways associated with inflammation and oxidative stress through the blockade of enzymatic activity.
32.	Garrigue et al., 2021 [32]	Resveratrol, a phytochemical, upon prolonged administration to animal models like rats showed a significant decrease in the expression of pro-inflammatory substances in the brain, thus enhancing neuro-cognitive function several fold.
33.	Sharma et al., 2022 [33]	The understanding of cellular senescence not only enables us to understand human physiology but also the implementation of dietary interventions to rejuvenate the immune system.
34.	Shahidi et al., 2018 [34]	The supplementation of omega-3 PUFAs has been evidenced to have beneficial effects against mental illnesses and age-related decline of immunity.
35.	Ali et al., 2022 [35]	A correlation between telomere lengthening and administration of omega-3 fatty acids was established through this study.
36.	Kiecolt-Glaser et al., 2012 [36]	The study observed a significant decrease in serum cytokines including IL-6 and a moderate decrease in TNF-alpha levels upon omega-3 supplementation which can ultimately have anti-aging effects.
37.	Challen et al., 2010 [37]	Different phenotypes of HSCs are stable under natural processes like aging but become unstable with competing HSCs. Furthermore, the varied responses of these phenotypes against TGF-beta1 signify possibilities of differential activation of HSC subtypes.
38.	Kovtonyuk et al., 2016 [38]	The correlation of hematopoietic aging and leukemogenesis with chronic inflammatory stimuli supports the theory of inflamm-aging and the elimination of these stimuli may aid in the preservation of HSCs and immune functions.
39.	Stahl et al., 2015 [39]	Reprogramming of cells and their conversion into iPSCs may serve as crucial in telomere elongation and the ultimate rejuvenation of the immune system with a possibility to improve the adult lifespan in the process.
40.	Al-Rashidi et al., 2022 [40]	A significant factor in disease pathogenesis is gut microflora dysbiosis, which can be shaped by the modulation of alterable environmental factors.
41.	Balcells et al., 2022 [41]	Marked reactivation of thymic functions was observed upon oral supplementation of probiotic strains and the removal of several negative stimuli like inflammatory mediators.
42.	Castro-Herrera et al., 2021 [42]	A significant increase in immune response to seasonal influenza vaccine was observed in the elderly upon administration of probiotic bacteria from the genus Lacticaseibacillus and Bifidobacterium.
43.	Costabile et al., 2017 [43]	The dietary inclusion of *L. rhamnosus* GG can be seen as a catalyst in improving the immune and microbial systems in the elderly as evidenced by a significant decrease in pro-inflammatory cytokines like IL-6.
44.	Aulin et al., 2021 [44]	Increased antibody concentrations and antigenic biomarkers in vaccines are effective in eliciting greater immune responses in the elderly due to the role of inflamm-aging coupled with cell-intrinsic defects in impaired vaccine responses.
45.	Pinti et al., 2016 [45]	The process of inflamm-aging can be reversed by remodeling of the immune response and modifications of the cell signaling pathways.
46.	de Punder et al., 2019 [46]	Telomerase activity is largely associated with factors like oxidative stress and inflammatory mediators and hence the modulation of these factors could be game-changing in the reversal of aging.
47.	Arem et al., 2015 [47]	Modest amounts of activity can benefit the inactive spectrum of the human population by postponing mortality and enhancing longevity.
48.	Walsh et al., 2011 [48]	Factors like personal hygiene, social distancing from sick people, and training methods like the use of appropriate recovery strategies have shown to be effective in reducing infection risk, hereby maintaining immune health.
49.	Walsh et al., 2011 [49]	Moderate amounts of exercise are known to reverse the effects of chronic stress, on the other hand, prolonged intense exercise may induce physiological pathways related to the deleterious effects of the same.

## Conclusions

Immune modulation studies may have been proven to be key to understanding aging. Positive health and longevity effects were demonstrated by several studies through lifestyle interventions like dietary restriction and physical activity. The key question now is the modulation of the inflamm-aging phenotypes through these interventions. We defined immunosenescence and its deleterious effects on health in the context of the immune system. We also focused on the treatment strategies, with a special emphasis on nutrition and its variable outcomes in the process of immunosenescence. The possibility of enhancing the efficacy and response of vaccinations for the elderly age group through immune-therapeutic interventions was also highlighted. Hence, we offer a compilation of various potential treatments aimed at mitigating immunosenescence.
